# Identifying Dementia research priority for Qatar national dementia research plan: A Cross-sectional Survey

**DOI:** 10.3126/nje.v14i2.69363

**Published:** 2024-09-02

**Authors:** Hanadi Al Hamad, Brijesh Sathian

**Affiliations:** 1 Geriatrics and long term care department, Rumailah Hospital, Hamad Medical Corporation, Doha, Qatar

**Keywords:** Demetia Research Priority, Dementia Risk Reduction, World Health Organization, Dementia, Qatar

## Abstract

**Background:**

The World Health Organization (WHO) published the Global Action Plan 2017-2025 seven years ago to address the dementia burden for those impacted, including persons living with dementia, their families, and health-care providers. There were seven action areas in the global action plan; the least achieved was action area seven (dementia research and innovation). The primary objective of the study was to assess the top 10 dementia research priorities among healthcare professionals, patients, caregivers, the public, and stakeholders to develop the Qatar National Dementia Research Plan.

**Methods:**

Convenience sampling was used in this cross-sectional survey. The study was conducted online with the involvement of HMC staff (physicians, nurses, and allied health staff) from all HMC facilities, patients and caregivers from Rumailah Hospital's Geriatric Department, and the public who attended the 2022 Advanced Dementia Research Conference. The survey was conducted during 22nd of October 2022 till April 31, 2024. Overall, 2000 participants provided their responses, which included health care professionals under HMC, including physicians, nurses, allied health staff, patients, caregivers, the public, and stakeholders in Qatar.

**Results:**

Dementia Risk Reduction (79%) was the top priority for the survey participants. The remaining nine priorities were the impact of early treatment (76%), health system capacity (73%), implementation of best practices for care (73), access to information and services post-diagnosis (71), caregiver support (70%), emotional well-being (67%), care provider education (65%), end-of-life care (65%), and non-drug approaches to managing symptoms (65%).

**Conclusion:**

The survey results clearly indicated that most participants ranked Dementia Risk Reduction as their top priority, indicating the essential focus on dementia prevention. These findings, together with goals such as early treatment, healthcare system capacity, and caregiver support, highlight the importance of an integrated, multidisciplinary approach to dementia management.

## Introduction

The World Health Organization (WHO) published the Global Action Plan 2017-2025 seven years ago to address the dementia burden for affected individuals, including persons living with dementia, their families, and healthcare providers [1]. The plan comprised seven action areas, with action area seven (dementia research and innovation) being the least accomplished. The implementation of dementia research that aligns with established goals and incorporates social and technological advancements has the potential to enhance the prevention, diagnosis, treatment, and care of individuals living with dementia. The WHO proposed actions for member states, including developing a national research agenda on dementia prevention, diagnosis, treatment, and care, in collaboration with academic institutions. Additionally, the plan aims to increase research capacity by engaging stakeholders, enhancing research infrastructure, improving researcher competency, and establishing centers of excellence for dementia research. However, with fewer than two years remaining to achieve this objective, progress remains insufficient. Nations worldwide face challenges in implementing practical measures to ensure that their policy objectives align with the Global Action Plan to Address Dementia; consequently, Alzheimer's Disease International has urged the WHO to extend the Global Action Plan's deadlines by four years, until 2029 [2,3].

Qatar's population continues to exhibit an aging trend, aligning broadly with other Arab countries, with the Gulf Cooperation Council (GCC) region projected to have the highest proportion of older individuals by 2050 [1,4]. Moreover, according to the United Nations’ demographic data for individuals over 60 years of age in 2017, Qatar has an estimated 4400 people living with dementia. This figure is expected to increase to more than 41,000 by 2050 [5]. Key risk factors for dementia, such as diabetes and obesity, are prevalent throughout the country [6-7].

The Qatar National Dementia Plan (QNDP) demonstrates the government's commitment to prioritizing dementia at the national level while also representing and realizing long-term efforts within the country to address its impact. Qatar joined the Global Dementia Observatory (GDO), a World Health Organization (WHO)-based platform dedicated to exchanging data on dementia research, policy, and service delivery with all member states [9], as one of only two pilot nations from the WHO Eastern Mediterranean Regional Office (EMRO) region [1]. Concurrently, the National Dementia Stakeholder Group has been established [5-11]. The Dementia Working Group analyzed the strengths, weaknesses, opportunities, and threats associated with dementia in Qatar. The QNDP aims to raise awareness, improve diagnosis, and support dementia patients, while also promoting research and innovation [5]. Ongoing multidisciplinary research is essential to understand and treat dementia, making it a policy priority. The primary objective of this study was to identify the top 10 dementia research priorities among healthcare professionals, patients, caregivers, the public, and stakeholders to develop the Qatar National Dementia Research Plan.

## Methodology

### Study design and participants

Convenience sampling was employed for this cross-sectional survey. The study was conducted online with the participation of HMC staff (physicians, nurses, and allied health professionals) from all HMC facilities, patients, and caregivers from Rumailah Hospital's Geriatric Department, and members of the public who attended the 2022 Advanced Dementia Research Conference. The survey was administered from October 22, 2022, to April 31, 2024. A total of 2000 participants provided responses, comprising healthcare professionals from HMC, including physicians, nurses, allied health staff, patients, caregivers, members of the public, and stakeholders in Qatar.

### Data collection:

The study was conducted online. The participants were invited to participate in the study through a research information sheet. An email containing research information sheets and links to the survey was disseminated to all healthcare professionals at the HMC facilities, including physicians, nurses, allied health staff, patients, caregivers, members of the public, and stakeholders. The questionnaire required an average of seven minutes to complete. Participation in the survey was entirely voluntary.

### Questionnaire design and validation The tool construction

The priorities mentioned were identified from existing research recommendations from the WHO GDO and established worldwide dementia research priorities [1, 12, 13]. The inclusion criterion was as follows: a novel research question within this domain applicable to the Qatar population. Technical recommendations such as the necessity for more rigorously conducted, larger-scale trials, alternative analyses, trial designs, or measurement methodologies were not included.

### Research Priorities

The following are the 15 research priorities set for Qatar for testing. All priorities were rated on a scale from 1 to 3. One was the top and three were the last.

Priority 1: Dementia Risk Reduction

Priority 2: Addressing stigma

Priority 3: Caregiver support

Priority 4: Clinical trials

Priority 5: Impact of early treatment

Priority 6: Care provider education

Priority 7: Dementia-friendly communities

Priority 8: End of life care

Priority 9: Health system capacity

Priority 10: Access to information and services post-diagnosis

Priority 11: Implementation of best practices for care

Priority 12: Non-drug approaches to managing symptoms

Priority 13: Nutritional interventions

Priority 14: Culturally adapted Screening tools

Priority 15: Emotional well-being

This validated tool can be adapted to any other country. Both Cronbach's alpha and McDonald's Omega showed a high reliability of 0.948.

### Inclusion criteria:

The inclusion criteria were health care professionals under HMC, including physicians, nurses, allied health staff, patients, caregivers, the public, and stakeholders in Qatar.

### Exclusion criteria:

Those who were unwilling to participate were excluded from the study.

### Sample size calculation

Considering the unknown percentage p=50% for dementia research priority, an absolute value of 2.5 at a confidence level of 95%, the required sample size was 1537. Accounting for a non-response rate of 30%, the total sample size required was 2000.

### Outcome Variable:

The outcome variables included 15 questions related to dementia research priorities.

### Explanatory variable:

Age, sex, and category were explanatory variables.

### Ethical committee approval

This study received approval from the Medical Research Centre, the Institutional Review Board of Hamad Medical Corporation (MRC-01-22-624). Participation in the study was voluntary and anonymous. The Institutional Review Board waived the requirement for written consent because it would be the only information to remove the anonymity of participation.

### Data Management and Statistical Analyses

The responses from the online survey were exported to Microsoft Excel. Descriptive and inferential statistics were used to analyze the data. Statistical significance was determined using the chi-square test, with a p-value < 0.05. All statistical analyses were conducted using R-4.3.2 for Windows software.

## Results

**Figure 1:**
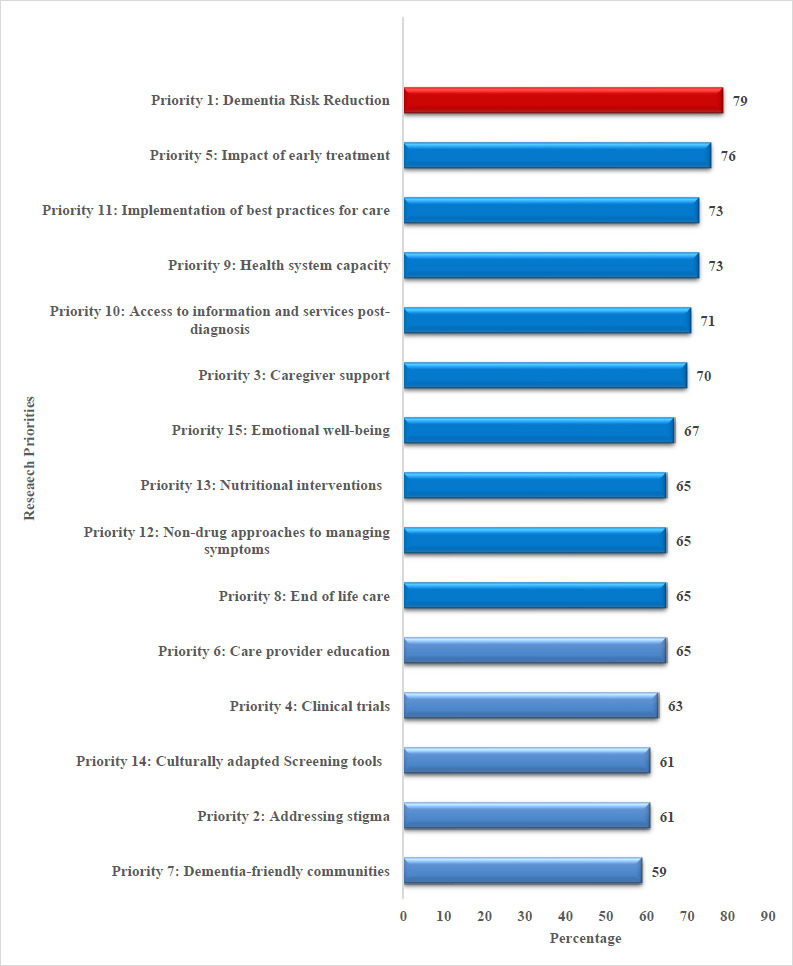
Dementia Research priority ranking Qatar

Of the 2000 patients, 62% were female. The sample comprised healthcare professionals (52%), caregivers (22%), patients (21%), members of the public (2%), researchers (2%), and stakeholders (1%). Figure 1 illustrates that dementia risk reduction (79%) was the primary priority of survey respondents. The remaining nine priorities were the impact of early treatment (76%), health system capacity (73%), implementation of best practices for care (73%), access to information and services post-diagnosis (71%), caregiver support (70%), emotional well-being (67%), care provider education (65%), end-of-life care (65%), and non-pharmacological approaches to managing symptoms (65%).

The analysis of dementia research priorities revealed substantial differences across various participant groups, highlighting the diverse perspectives within the community. While both gender and stakeholder categories demonstrated agreement on the importance of numerous research areas, notable disparities emerged in the prioritization of specific topics.

Care provider education (69.5% vs. 62.8%, p=0.006), access to information and services post-diagnosis (74.0% vs. 68.5%, p=0.032), implementation of best practices for care (75.8% vs. 71.5%, p=0.033), and non-drug approaches to symptom management (68.2% vs. 62.8%, p=0.034) were statistically significant with respect to gender. However, all priorities exhibited statistical significance in the category-wise comparisons (p <0.01).

In contrast, the category-wise analysis revealed that caregivers and patients consistently ranked dementia risk reduction and priority and addressed stigma higher than healthcare professionals and the public. Care provider education was also more frequently ranked as the top priority by patients (82.1%) and caregivers (79.0%) than by healthcare professionals (52.7%) and the public (58.8%). These subgroup analyses underscore the significance of considering the distinct needs and experiences of different groups when prioritizing dementia research.

## Discussion

This study aimed to identify and rank the top dementia research priorities among health care professionals, patients, caregivers, the public, and other stakeholders in Qatar. The findings highlight significant gender and category-wise differences in the prioritization of dementia research topics, underscoring the importance of a tailored approach in setting research agendas that reflect the diverse perspectives of various stakeholders.

### Key Findings and Implications

The findings of this study indicate that dementia risk reduction is the primary concern of survey respondents. This prioritization may be attributed to the efficacy of dementia risk-reduction awareness initiatives in Qatar [14-18]. The nine subsequent priorities identified were the impact of early intervention, health system capacity, implementation of optimal care practices, access to post-diagnostic information and services, caregiver support, emotional well-being, care provider education, end-of-life care, and non-pharmacological approaches to symptom management.

In contrast, a recent systematic review of ten studies elucidated critical targets for future dementia research agendas [19]. The majority of published prioritization exercises identified caregivers and support as the primary objectives for dementia research, with less emphasis on pharmacological interventions and therapies. Notably, no studies have included individuals at risk for dementia development (e.g., healthy older adults, those with family history, or genetic predisposition) as stakeholders. This omission likely accounted for the low priority assigned to prevention-based research. Additionally, a European scoping review revealed that dementia care objectives differed from dementia research goals; however, the unifying theme was the necessity to comprehend the biopsychosocial components of dementia rather than adopting a purely biological approach [20, 21]. Research priorities exhibit regional variations, particularly between low-income and high-income countries [20]. Consequently, priority-setting exercises conducted in one context may not be applicable or relevant to another.

The First WHO Ministerial Conference on Global Action Against Dementia, held in 2015, emphasized the necessity for collaborative research to mitigate the global burden of dementia [21]. A priority-setting process identified six primary research areas: dementia prevention, risk identification and reduction, care delivery, diagnosis, biomarkers, therapy development, disease etiology, and public awareness. These objectives should be mapped against the global dementia research landscape in order to inform policymakers, funding bodies, and researchers. Stakeholders, including the WHO, member states, and civil society organizations, should monitor their research expenditure and progress.

The Lancet Commission on Dementia 2024 updates present novel findings on dementia prevention, intervention, and care [22]. The prevalence of dementia is increasing due to increased longevity despite a decline in age-specific incidence in high-income nations. This report underscores the importance of identifying and implementing prevention strategies, reducing vascular damage, and addressing risk factors, including low educational attainment, hearing impairment, hypertension, smoking, obesity, depression, diabetes, alcohol consumption, traumatic brain injury, air pollution, and social isolation.

### Limitations and Strengths

A significant limitation of this study was the use of continence sampling. An additional limitation is the disparity in sample sizes when comparing categories. The primary strength of this study lies in the inclusion of a diverse cohort of participants, which ensures that the identified priorities reflect a broad spectrum of perspectives.

### Implications for Future Research and Policy

These research priorities should inform the development of Qatar's national dementia research strategy. Policymakers and researchers should consider the diverse interests of multiple stakeholder groups to ensure that the research agenda is comprehensive and pertinent to societal needs. Moreover, substantial gender and category disparities in prioritization should prompt further investigation into the underlying causes of these variations, potentially leading to more targeted research initiatives.

As Qatar has to implement its national dementia research plan encompassing caregivers, patients, and healthcare professionals, the perspectives on research activities will be crucial for establishing effective, evidence-based interventions. This approach will also contribute to the broader objectives of WHO's Global Action Plan on the Public Health Response to Dementia, which emphasizes the necessity for inclusive and participatory research methodologies.

## Conclusion

The survey results unequivocally demonstrated that the majority of participants identified dementia risk reduction as their primary concern, indicating the critical importance of dementia prevention. These findings, in conjunction with objectives such as early intervention, healthcare system capacity, and caregiver support, underscore the necessity for an integrated, multidisciplinary approach to dementia management.

### Future scope of the study

There is a need of qualitative stuy to understand the indepth need of this group.

### What is already known on this topic?

There are dementia research priorities available for other few countries around the globe.

### What this study adds:

This study provides a comprehensive analysis of dementia research priorities of Qatar

## Figures and Tables

**Table 1:** Gender-wise crosstabs of research priorities

Priorities	Gender			P Value
	Female n (%)	Male n (%)	Total n (%)	
**Age**	45.2 ± S.D 12.9	49.4 ± S.D 14.3		
**Priority 1: Dementia Risk Reduction**				0.66
**1**	961 (77.3%)	618 (81.6%)	1579 (79.0%)	
**2**	220 (17.7%)	106 (14.0%)	326 (16.3%)	
**3**	62 (5.0%)	33 (4.4%)	95 (4.8%)	
**Priority 2: Addressing stigma**				0.858
**1**	756 (60.8%)	469 (62.0%)	1225 (61.3%)	
**2**	401 (32.3%)	239 (31.6%)	640 (32.0%)	
**3**	86 (6.9%)	49 (6.5%)	135 (6.8%)	
**Priority 3: Caregiver support**				0.92
**1**	866 (69.7%)	535 (70.7%)	1401 (70.0%)	
**2**	293 (23.6%)	175 (23.1%)	468 (23.4%)	
**3**	79 (6.4%)	45 (5.9%)	124 (6.2%)	
**Priority 4: Clinical trials**				0.227
**1**	764 (61.5%)	498 (65.8)	1262 (63.1%)	
**2**	377 (30.3%)	210 (27.7%)	587 (29.3%)	
**3**	96 (7.7%)	46 (6.1%)	142 (7.1%)	
**Priority 5: Impact of early treatment**				0.143
**1**	925 (74.4)	588 (77.7%)	1513 (75.6%)	
**2**	246 (19.8%)	136 (18.0%)	382 (19.1%)	
**3**	69 (5.6%)	29 (3.8%)	98 (4.9%)	
**Priority 6: Care provider education**				0.006*
**1**	781 (62.8%)	526 (69.5%)	1307 (65.3%)	
**2**	386 (31.1%)	189 (25.0%)	575 (28.7%)	
**3**	71 (5.7%)	42 (5.5%)	113 (5.7)	
**Priority 7: Dementia-friendly communities**				0.426
**1**	735 (59.1%)	451 (59.6%)	1186 (59.3%)	
**2**	380 (30.6%)	224 (29.6%)	604 (30.2%)	
**3**	121 (9.7%)	81 (10.7%)	202 (10.1%)	
**Priority 8: End of life care**				0.266
**1**	795 (64.0%)	511 (67.5%)	1306 (65.3%)	
**2**	334 (26.9%)	185 (24.4%)	519 (25.9%)	
**3**	108 (8.7%)	60 (7.9%)	168 (8.4%)	
**Priority 9: Health system capacity**				0.29
**1**	879 (70.7%)	580 (76.6%)	1459 (73.0%)	
**2**	274 (22.0%)	133 (17.6%)	407 (20.3%)	
**3**	85 (22.0%)	43 (5.7%)	128 (6.4%)	
**Priority 10: Access to information and services post-diagnosis**				0.032*
**1**	852 (68.5%)	560 (74.0%)	1412 (70.6%)	
**2**	294 (23.7%)	159 (21.0%)	453 (22.7%)	
**3**	89 (7.2%)	35 (4.6%)	124 (6.2%)	
**Priority 11: Implementation of best practices for care**				0.033*
**1**	889 (71.5%)	574 (75.8%)	1463 (73.2%)	
**2**	259 (20.8%)	147 (19.4%)	406 (20.3%)	
**3**	87 (7.0%)	35 (4.6%)	122 (6.1%)	
**Priority 12: Non-drug approaches to managing symptoms**				0.034*
**1**	781(62.8%)	516(68.2%)	1297 (64.8%)	
**2**	357 (28.7%)	192 (25.4%)	549 (27.5%)	
**3**	101(8.1%)	44 (5.8%)	145 (7.2%)	
**Priority 13: Nutritional interventions**				0.32
**1**	790 (63.6%)	511 (67.5%)	1301 (65.0%)	
**2**	356 (28.6%)	189 (25.0%)	545 (27.3%)	
**3**	92 (7.4%)	54 (7.1%)	146 (7.3%)	
**Priority 14: Culturally adapted Screening tools**				0.182
**1**	738 (59.4%)	483 (63.8%)	1221 (61.1%)	
**2**	379 (30.5%)	214 (28.3%)	593 (29.6%)	
**3**	123 (9.9%)	59 (7.8%)	182 (9.1%)	
**Priority 15: Emotional well-being**				0.442
**1**	824 (66.3%)	512 (67.6%)	1336 (66.8%)	
**2**	330 (26.5%)	192 (25.4%)	522 (26.1%)	
**3**	87 (7.0%)	49 (6.5%)	136 (6.8%)	

*Chi-square p value <0.05

**Table 2:** Category-wise crosstabs of research priorities

Priorities	Categories (n %)						P value
	Caregivers	HCP	Patient	Public	Researcher	Stakeholder	
**Age**	41.8 ± S.D 7.7	40.9 ± S.D 8.4	67.5 ± S.D 7.6	37.3 ± S.D 9.8	38.5 ± S.D 10.0	37.1 ± S.D 3.4	
**Priority 1: Dementia Risk Reduction**							<0.001*
**1**	424 (94.6%)	707 (67.9%)	395 (90.6%)	24 (70.6%)	23 (69.7%)	6 (85.7%)	
**2**	22 (4.9%)	255 (24.5%)	34 (7.8%)	8 (23.5%)	6 (18.2%)	1 (14.3%)	
**3**	2 (0.4%)	80 (7.7%)	7 (1.6%)	2 (5.9%)	4 (12.1%)	0 (0.0%)	
**Priority 2: Addressing stigma**							<0.001*
**1**	315 (70.3%)	540 (51.8%)	336 (77.1%)	17 (50.0%)	14 (42.4%)	3 (42.9%)	
**2**	126 (28.1%)	393 (37.7%)	89 (20.4%)	14 (41.2%)	14 (42.4%)	4 (57.1%)	
**3**	7 (1.6%)	109 (10.5%)	11 (2.5%)	3 (8.8%)	5 (15.2%)	0 (0.0%)	
**Priority 3: Caregiver support**							<0.001*
**1**	360 (80.4%)	637 (61.1%)	357 (81.9%)	19 (55.9%)	22 (66.7%)	6 (85.7%)	
**2**	83 (18.5%)	292 (28.0%)	73 (16.7%)	11 (32.4%)	8 (24.2%)	1 (14.3%)	
**3**	5 (1.1%)	107 (10.3%)	6 (1.4%)	4 (11.8%)	2 (6.1%)	0 (0.0%)	
**Priority 4: Clinical trials**							<0.001*
**1**	322 (71.9%)	568 (54.5%)	324 (74.3%)	21 (61.8%)	22 (66.7%)	5 (71.4%)	
**2**	116 (25.9%)	353 (33.9%)	101 (23.2%)	8 (23.5%)	7 (21.2%)	2 (28.6%)	
**3**	10 (2.2%)	112 (10.7%)	11 (2.5%)	5 (14.7%)	4 (12.1%)	0 (0.0%)	
**Priority 5: Impact of early treatment**							<0.001*
**1**	393 (87.7%)	696 (66.8%)	371 (85.1%)	23 (67.6%)	24 (72.7%)	6 (85.7%)	
**2**	47 (10.5%)	259 (24.9%)	61 (14.0%)	8 (23.5%)	6 (18.2%)	1 (14.3%)	
**3**	8 (1.8%)	81 (7.8%)	4 (0.9%)	3 (8.8%)	2 (6.1%)	0 (0.0%)	
**Priority 6: Care provider education**							<0.001*
**1**	354 (79.0%)	549 (52.7%)	358 (82.1%)	20 (58.8%)	22 (66.7%)	4 (57.1%)	
**2**	88 (19.6%)	392 (37.6%)	72 (16.5%)	12 (35.3%)	8 (24.2%)	3 (42.9%)	
**3**	6 (1.3%)	97 (9.3%)	5 (1.1%)	2 (5.9%)	3 (9.1%)	0 (0.0%)	
**Priority 7: Dementia-friendly communities**							<0.001*
**1**	292 (65.2%)	542 (52.0%)	311 (71.3%)	23 (67.6%)	14 (42.4%)	4 (57.1%)	
**2**	127 (28.3%)	347 (33.3%)	107 (24.5%)	8 (23.5%)	12 (36.4%)	3 (42.9%)	
**3**	29 (6.5%)	147 (14.1%)	18 (4.1%)	2 (5.9%)	6 (18.2%)	0 (0.0%)	
**Priority 8: End of life care**							<0.001*
**1**	345 (77.0%)	56 (53.8%)	350 (80.3%)	23 (67.6%)	23 (69.7%)	4 (57.1%)	
**2**	86 (77.0%)	346 (53.8%)	73 (80.3%)	5 (14.7%)	6 (18.2%)	3 (42.9%)	
**3**	15 (3.3%)	130 (12.5%)	13 (3.0%)	6 (17.6%)	4 (12.1%)	0 (0.0%)	
**Priority 9: Health system capacity**							<0.001*
**1**	411 (91.7%)	616 (59.1%)	386 (88.5%)	20 (58.8%)	22 (66.7%)	4 (57.1%)	
**2**	32 (7.1%)	309 (29.7%)	46 (10.6%)	10 (29.4%)	7 (21.2%)	3 (42.9%)	
**3**	5 (1.1%)	113 (10.8%)	3 (0.7%)	4 (11.8%)	3 (9.1%)	0 (0.0%)	
**Priority 10: Access to information and services post-diagnosis**							<0.001*
**1**	400 (89.3%)	578 (55.5%)	384 (88.1%)	24 (70.6%)	23 (69.7%)	3 (42.9%)	
**2**	42 (9.4%)	347 (33.3%)	47 (10.8%)	6 (17.6%)	7 (21.2%)	4 (57.1%)	
**3**	6 (1.3%)	107 (10.3%)	5 (1.1%)	4 (11.8%)	2 (6.1%))	0 (0.0%)	
**Priority 11: Implementation of best practices for care**							<0.001*
**1**	399 (89.1%)	630 (60.5%)	384 (88.1%)	23 (67.6%)	22 (66.7%)	5 (71.4%)	
**2**	43 (9.6%)	299 (28.7%)	47 (10.8%)	7 (20.6%)	8 (24.2%)	2 (28.6%)	
**3**	6 (1.3%)	104 (10.0%)	5 (1.1%)	4 (11.8%)	3 (9.1%)	0 (0.0%)	
**Priority 12: Non-drug approaches to managing symptoms**							<0.001*
**1**	350 (78.1%)	568 (54.5%)	335 (76.8%)	24 (70.6%)	16 (48.5%)	4 (57.1%)	
**2**	83 (18.5%)	352 (33.8%)	90 (20.6%)	8 (23.5%)	13 (39.4%)	3 (42.9%)	
**3**	11 (2.5%)	118 (11.3%)	10 (2.3%)	2 (5.9%)	4 (12.1%)	0 (0.0%)	
**Priority 13: Nutritional interventions**							<0.001*
**1**	357 (79.7%)	553 (53.1%)	350 (80.3%)	21 (61.8%)	17 (51.5%)	3 (42.9%)	
**2**	81 (18.1%)	367 (35.2%)	74 (17.0%)	7 (20.6%)	12 (36.4%)	4 (57.1%)	
**3**	10 (2.2%)	115(11)	12 (2.8%)	5 (14.7%)	4 (12.1%)	0 (0.0%)	
**Priority 14: Culturally adapted Screening tools**							<0.001*
**1**	327 (73.0%)	520 (49.9%)	333 (76.4%)	19 (55.9%)	18 (54.5%)	4 (57.1%)	
**2**	97 (21.7%)	389 (37.3%)	85 (19.5%)	10 (29.4%)	9 (27.3%)	3 (42.9%)	
**3**	24 (5.4%)	130 (12.5%)	17 (3.9%)	5 (14.7%)	6 (18.2%)	0 (0.0%)	
**Priority 15: Emotional well-being**							<0.001*
**1**	338 (75.4%)	612 (58.7%)	342 (78.4%)	21 (61.8%)	18 (54.5%)	5 (71.4%)	
**2**	94 (21.0%)	323 (31.0%)	83 (19.0%)	9 (26.5%)	11 (33.3%)	2 (28.6%)	
**3**	14 (3.1%)	103 (9.9%)	11 (2.5%)	4 (11.8%)	4 (12.1%)	0 (0.0%)	

*Chi-square p value <0.05
